# Supplementary material for: Simulating the Effects of Different Textural Soils and N Management on Maize Yield, N Fates, and Water and N Use Efficiencies in Northeast China
Source: Plants (Basel). 2022 Dec 1;11(23):3338. doi: 10.3390/plants11233338 (PMC9741021; doi:10.3390/plants11233338)
Supplement: Supplementary file 1 [file plants-11-03338-s001.zip › plants-2031718-supplementary.pdf]

Supplementary Materials

Simulating the Effects of Different Textural Soils and N Management on Maize Yield, N Fates, and Water and N Use Efficiencies in Northeast China

Fanchao Meng [1], Kelin Hu [2,*], Puyu Feng [2], Guozhong Feng [1] and Qiang Gao [1,*]

1 College of Resources and Environmental Sciences, Key Laboratory of Straw Comprehensive Utilization and Black Soil Conservation, Jilin Agricultural University, Changchun 130118, China
2 College of Land Science and Technology, Key Laboratory of Arable Land Conservation (North China),
Ministry of Agriculture and Rural Affairs, China Agricultural University, Beijing 100193, China
* Correspondence: hukel@cau.edu.cn (K.H.); gyt199962@126.com (Q.G.)

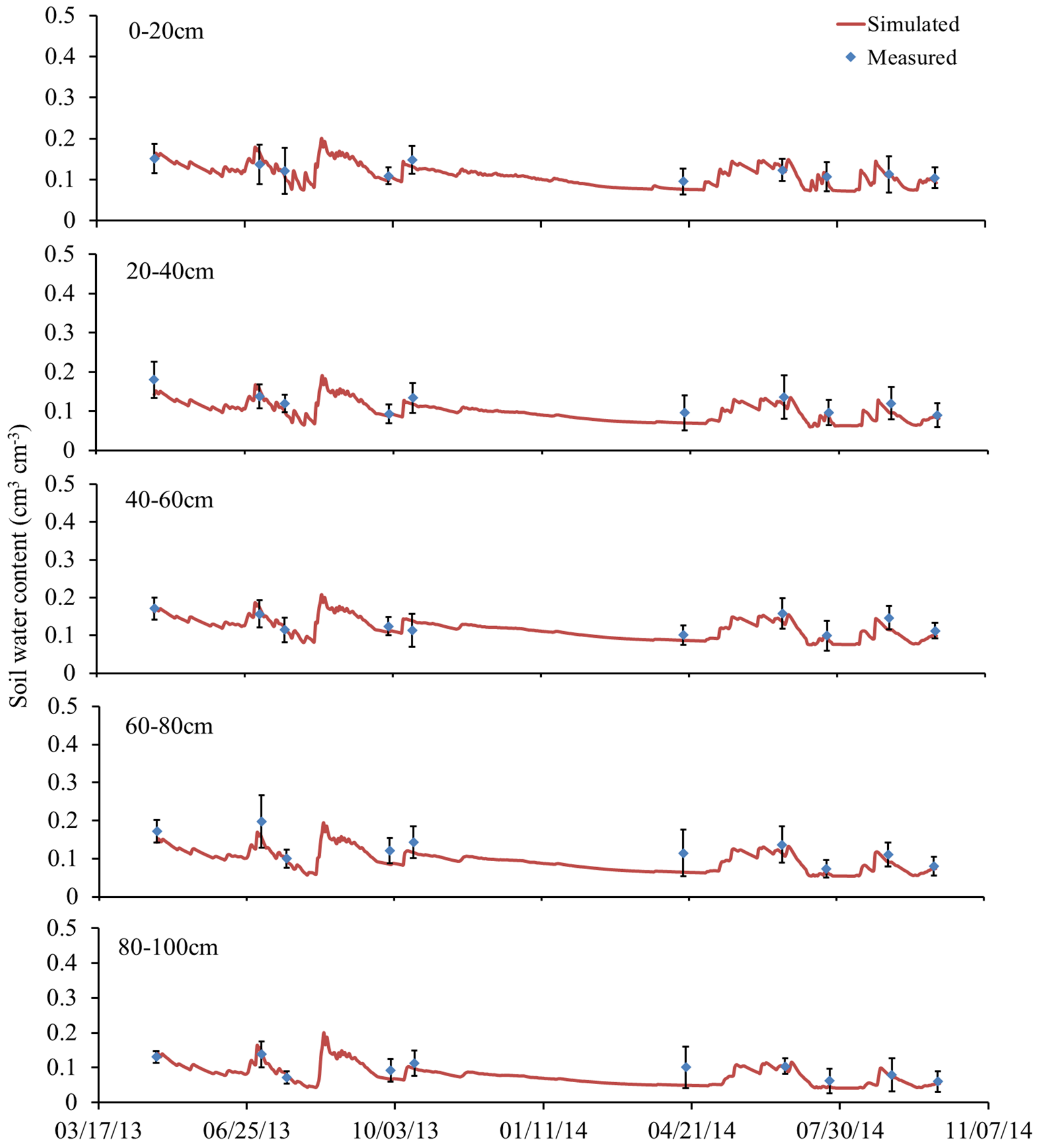

Figure S1. Comparison of simulated and measured soil water content at different depths under N312 treatment in Sandy loam soil.

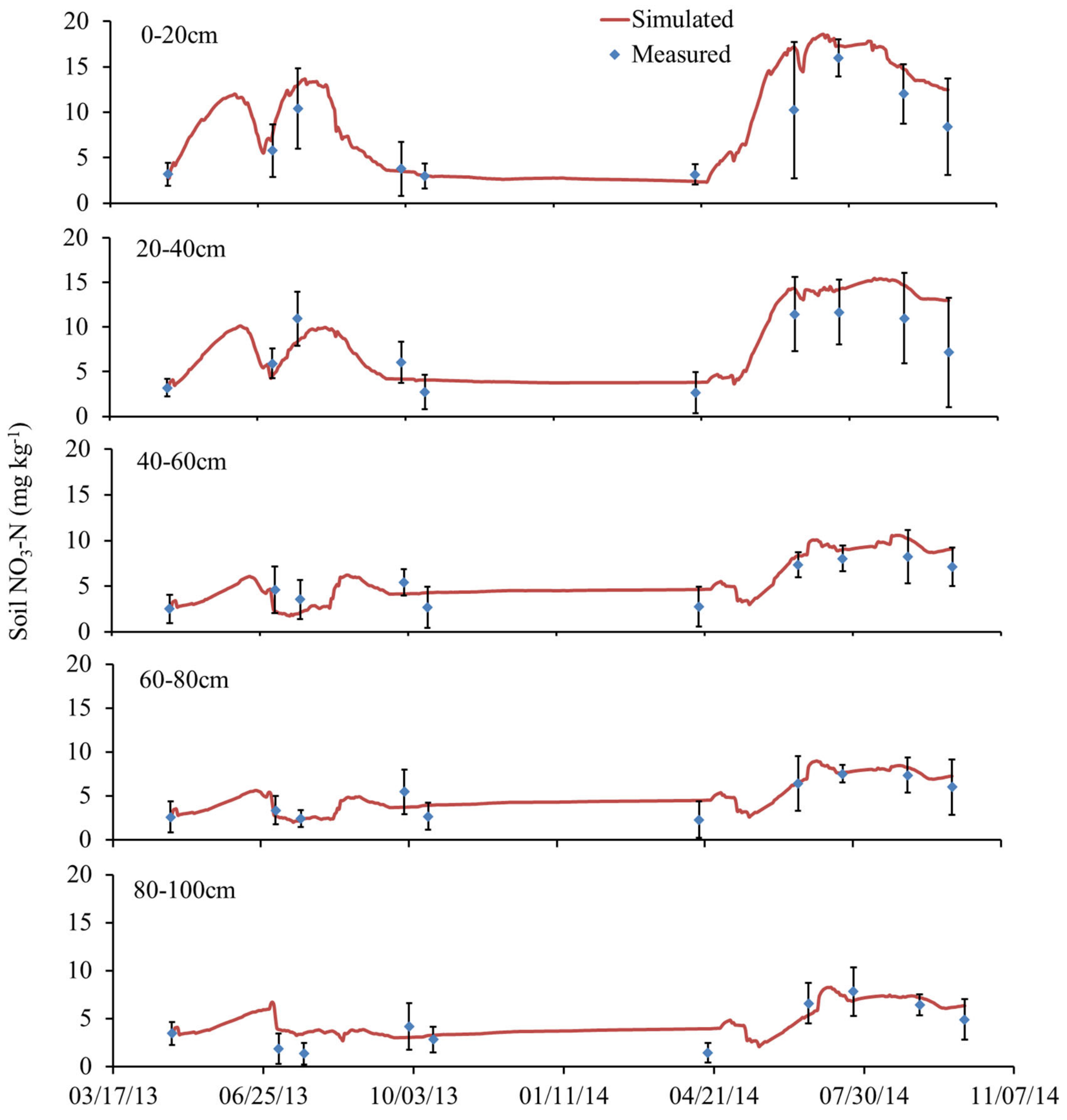

Figure S2. Comparison of simulated and measured soil nitrate concentration at different depths under N312 treatment in Sandy loam soil.

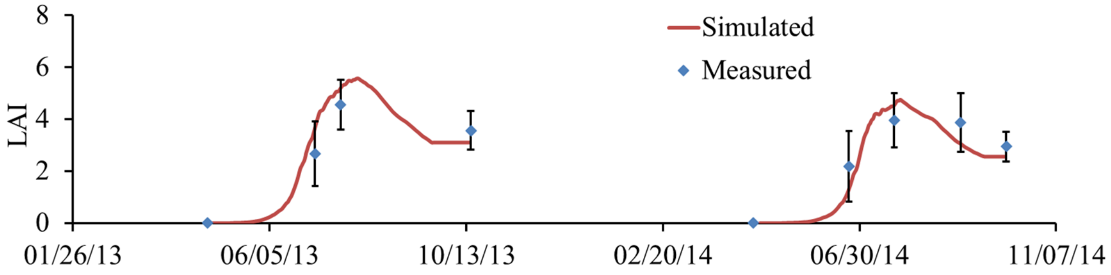

Figure S3. Comparison of measured and simulated leaf area index (LAI) under N312 treatment in Sandy loam soil.

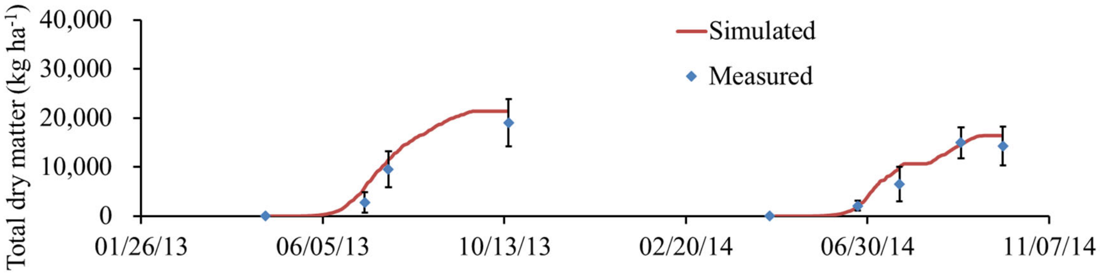

Figure S4. Comparison of measured and simulated total dry matter (TDM) under N312 treatment in Sandy loam soil.

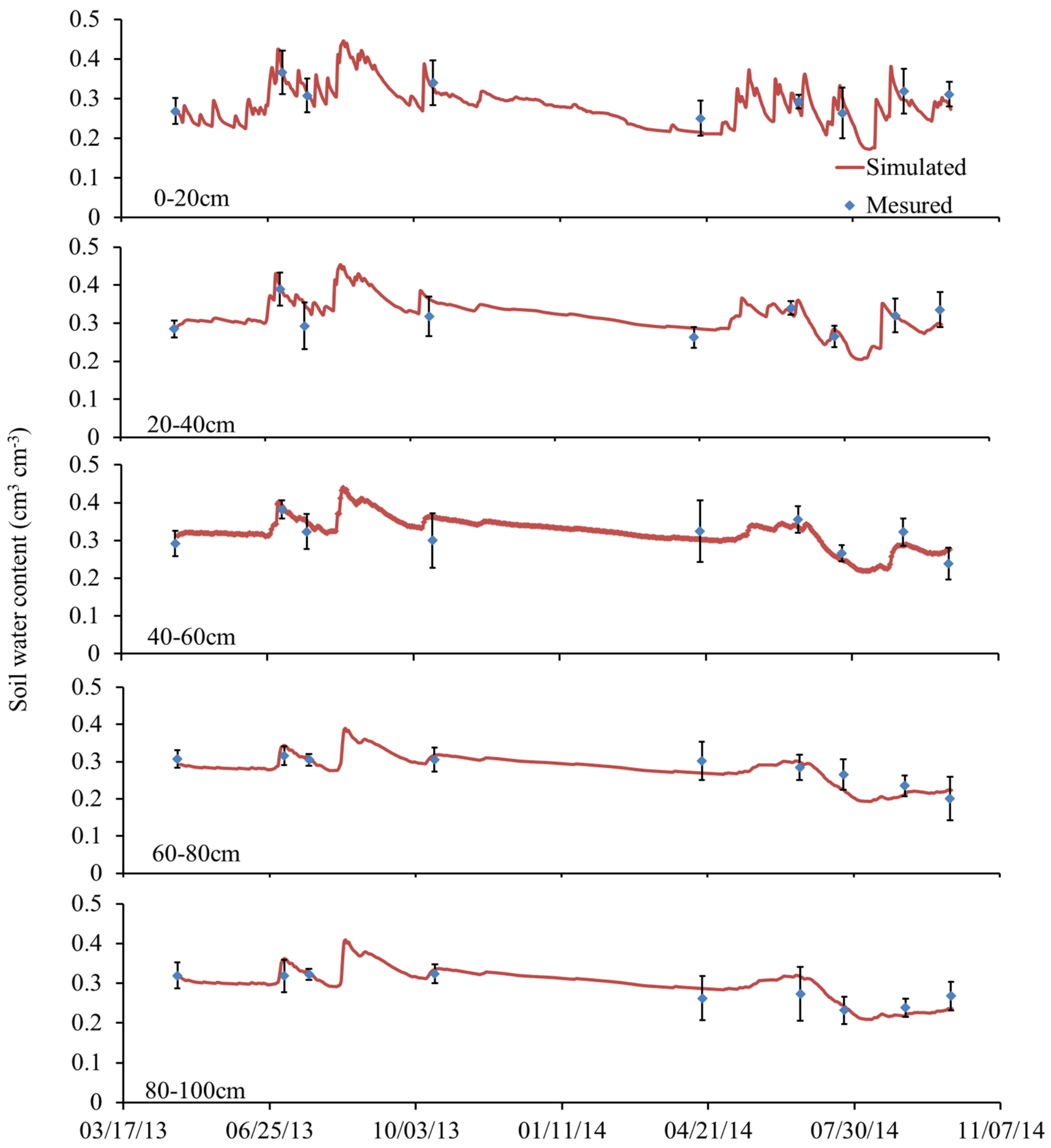

Figure S5. Comparison of simulated and measured soil water content at different depths under N240 treatment in loamy clay soil.

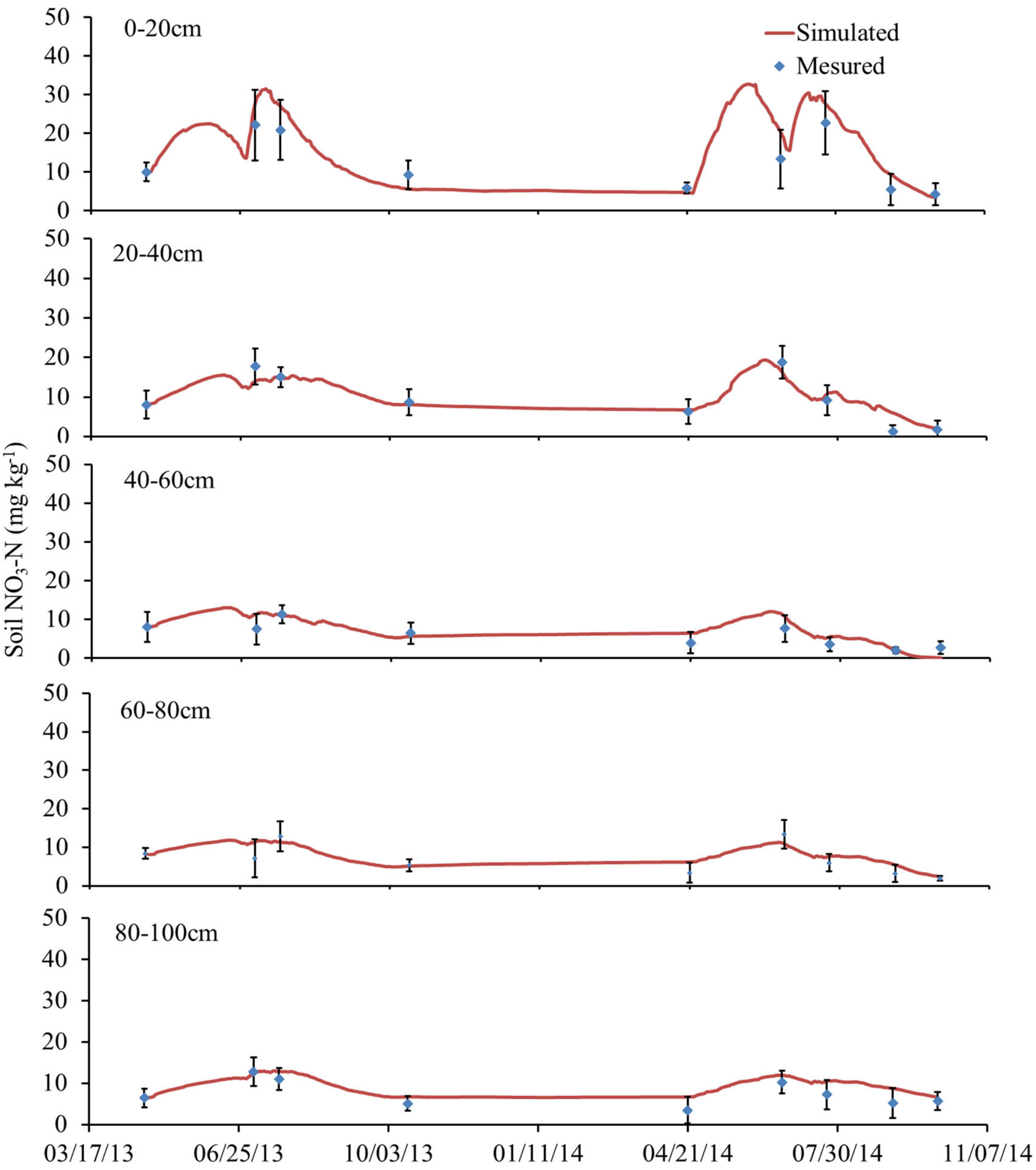

Figure S6. Comparison of simulated and measured soil nitrate concentration at different depths under N240 treatment in loamy clay soil.

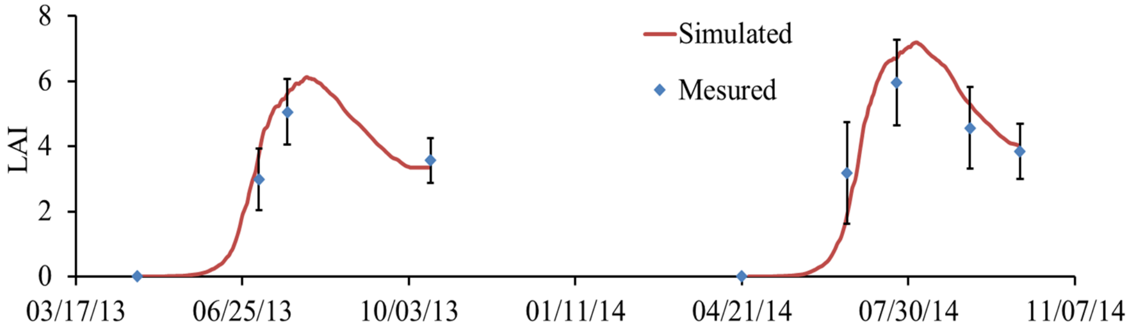

Figure S7. Comparison of measured and simulated leaf area index (LAI) under N240 treatment in loamy clay soil.

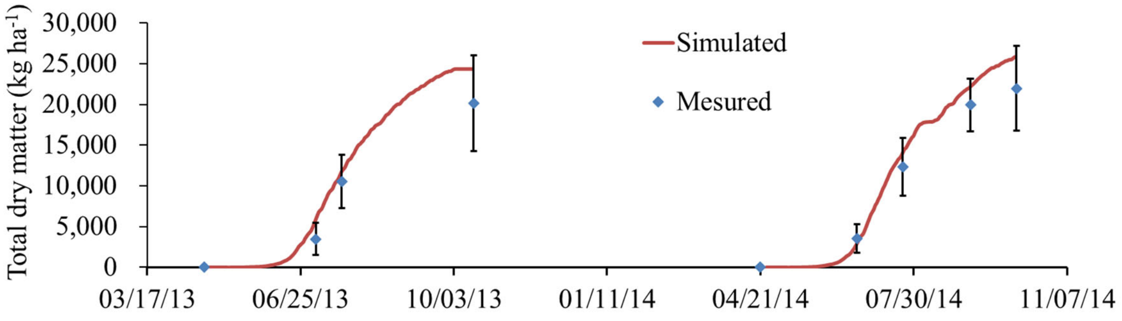

Figure S8. Comparison of measured and simulated total dry matter (TDM) under N240 treatment in loamy clay soil.

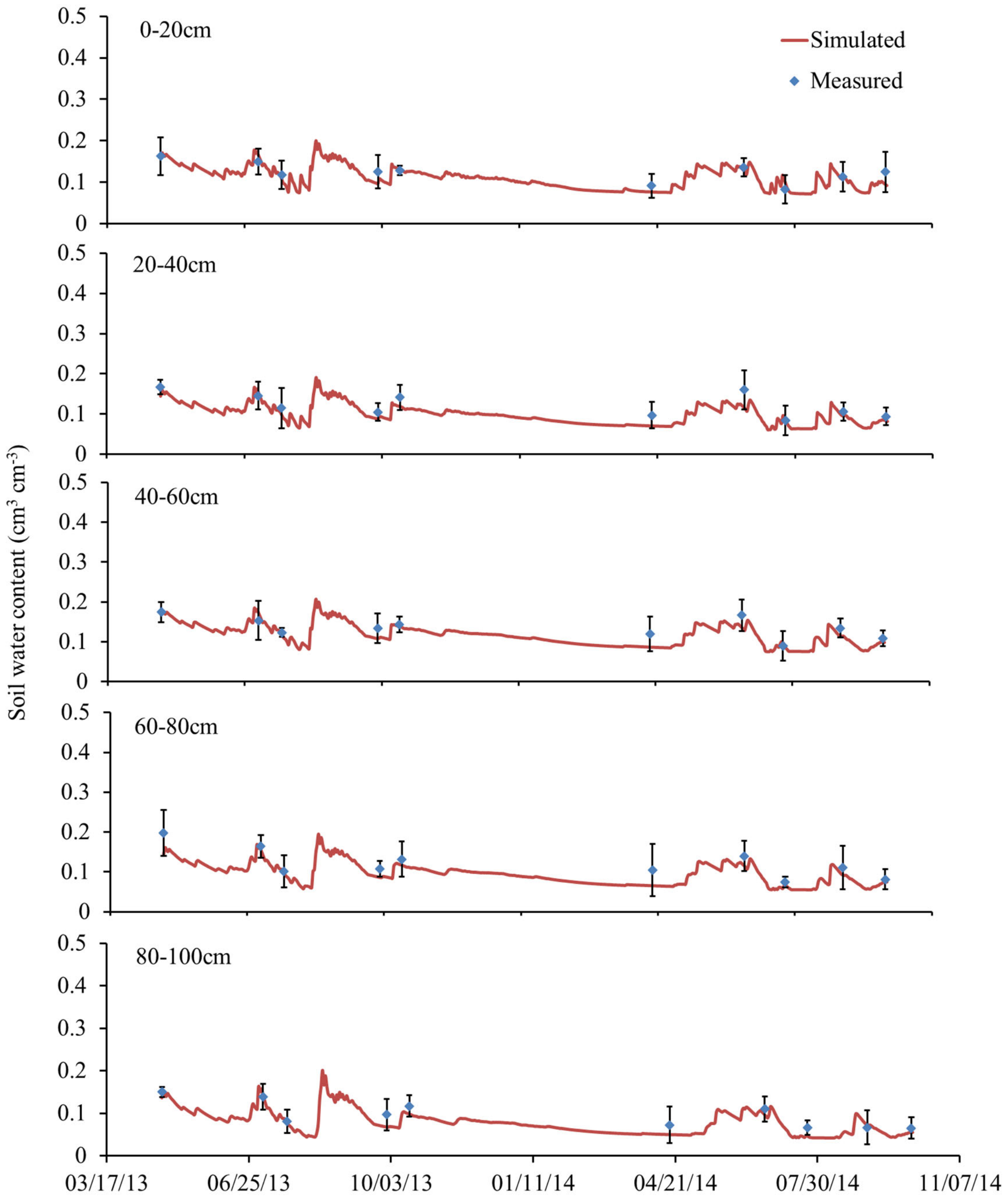

Figure S9. Comparison of simulated and measured soil water content at different depths under N240 treatment in Sandy loam soil.

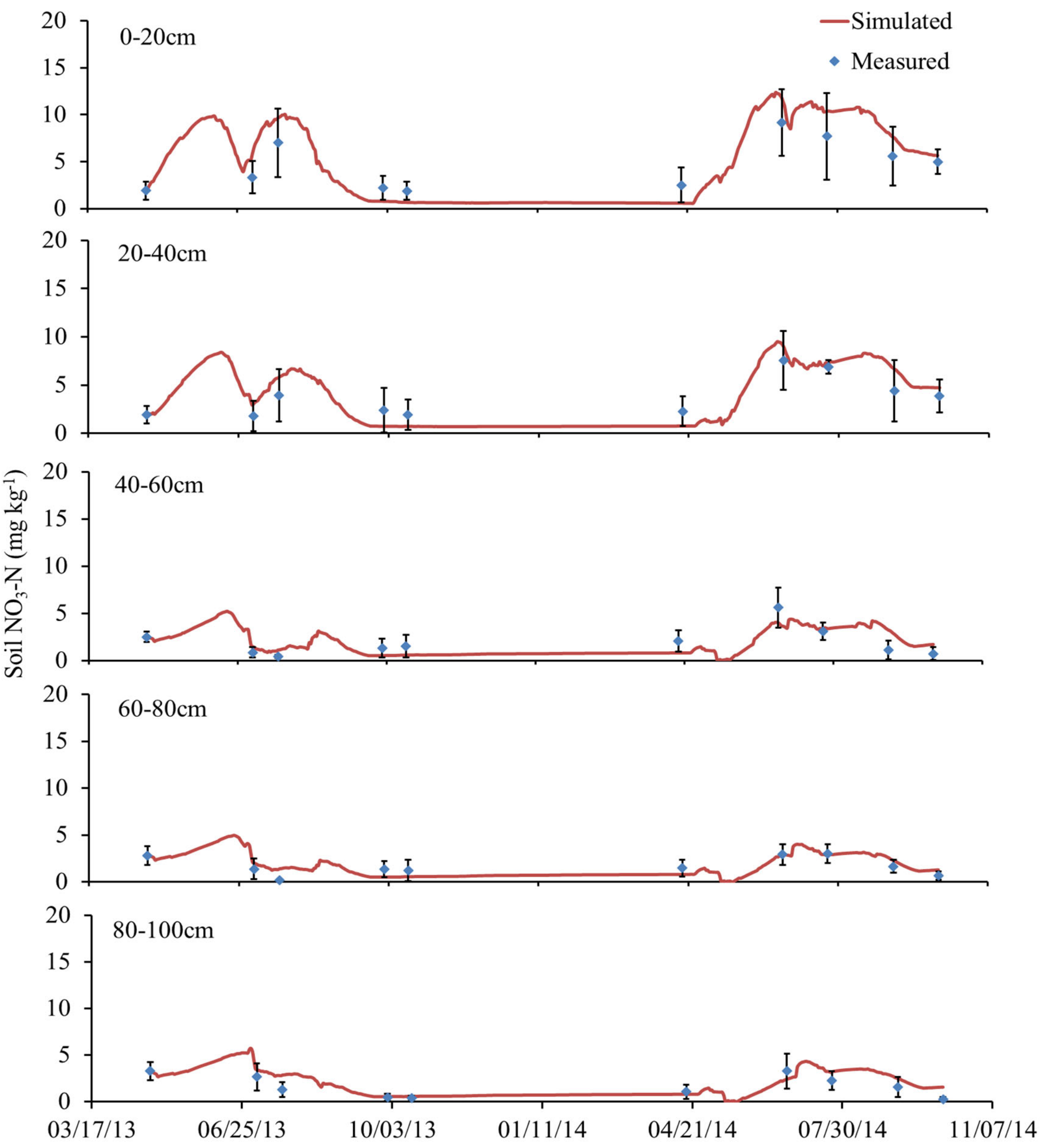

Figure S10. Comparison of simulated and measured soil nitrate concentration at different depths under N240 treatment in Sandy loam soil.

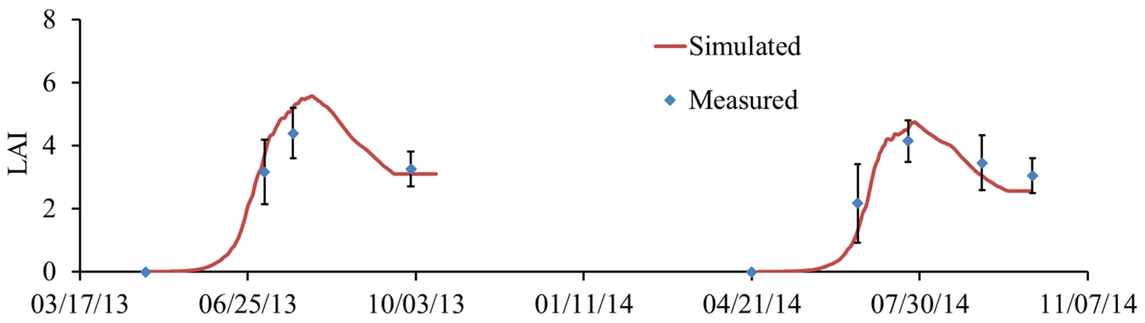

Figure S11. Comparison of measured and simulated leaf area index (LAI) under N240 treatment in Sandy loam soil

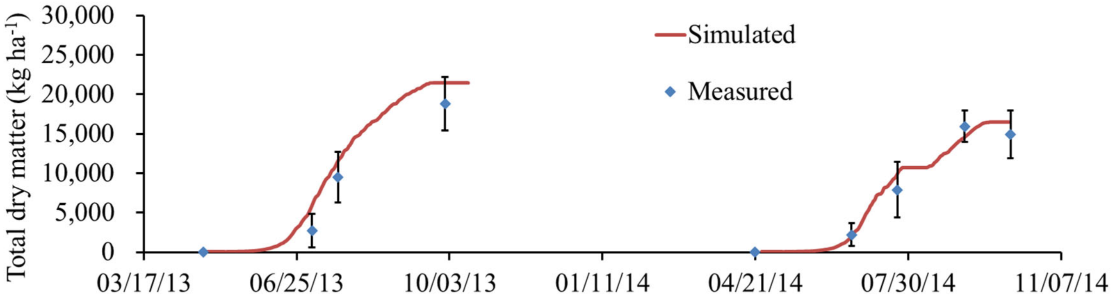

Figure S12. Comparison of measured and simulated total dry matter (TDM) under N240 treatment in Sandy loam soil.

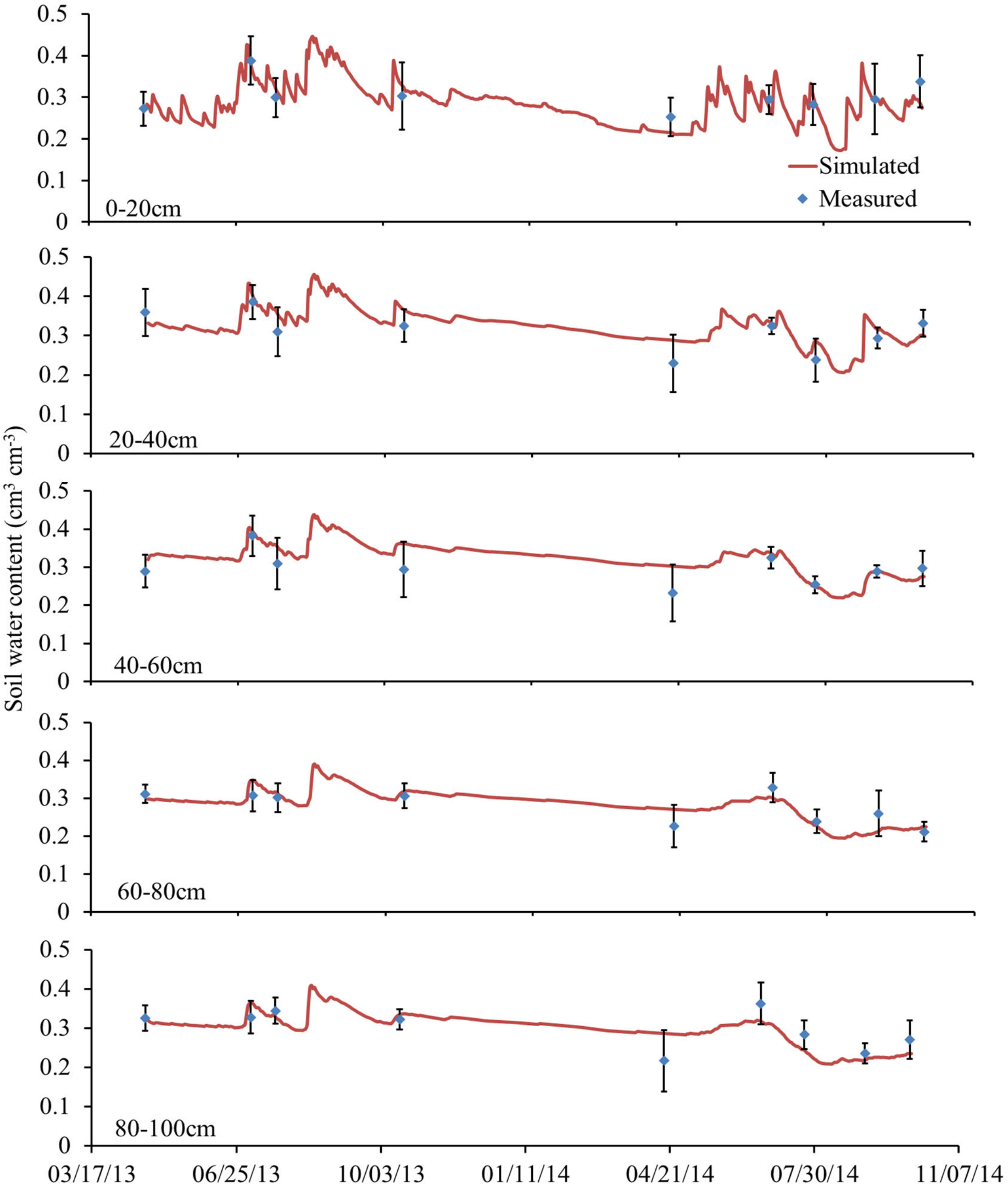

Figure S13. Comparison of simulated and measured soil water content at different depths under N168 treatment in loamy clay soil.

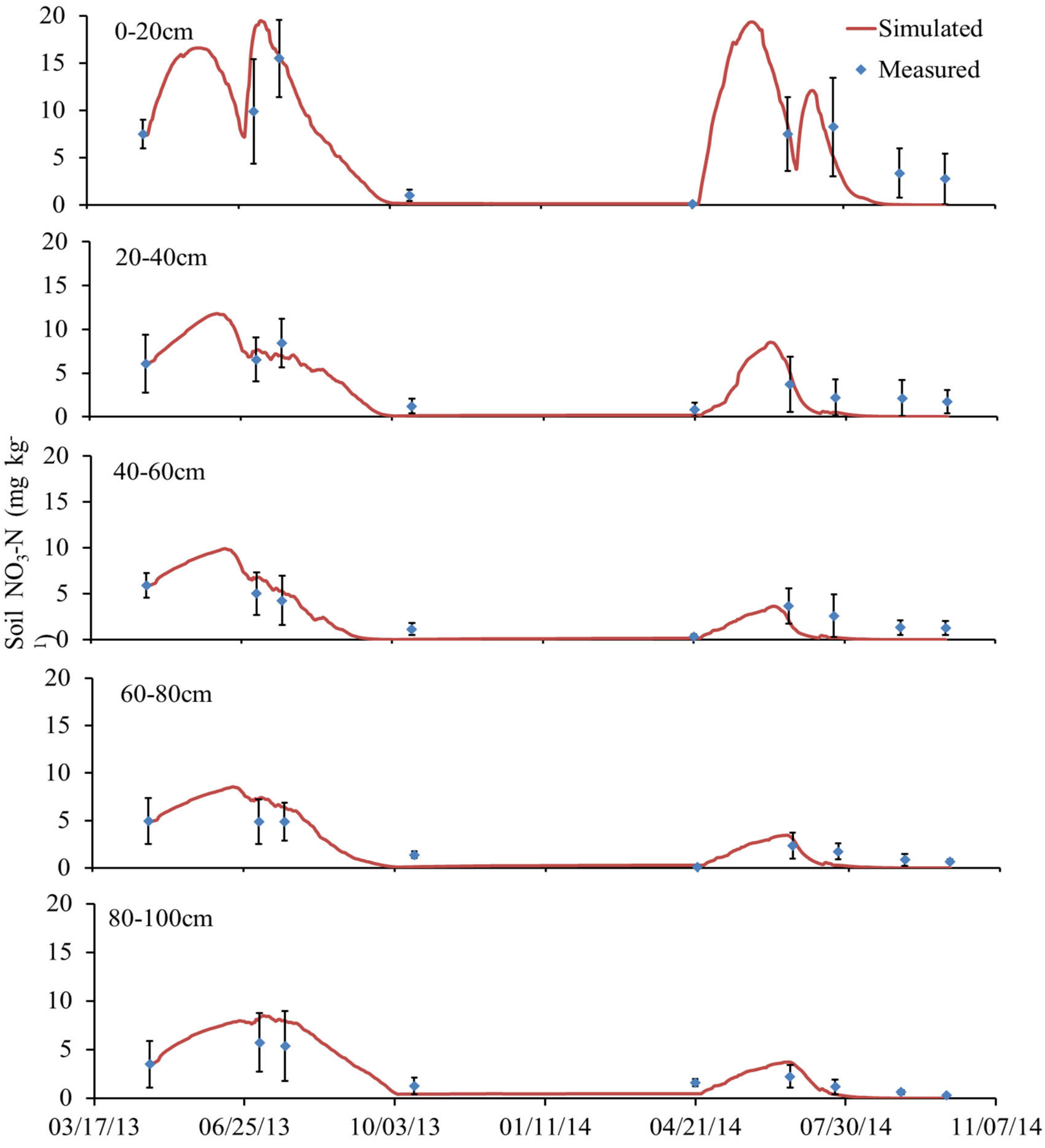

FigureS14. Comparison of simulated and measured soil nitrate concentration at different depths under N168 treatment in loamy clay soil.

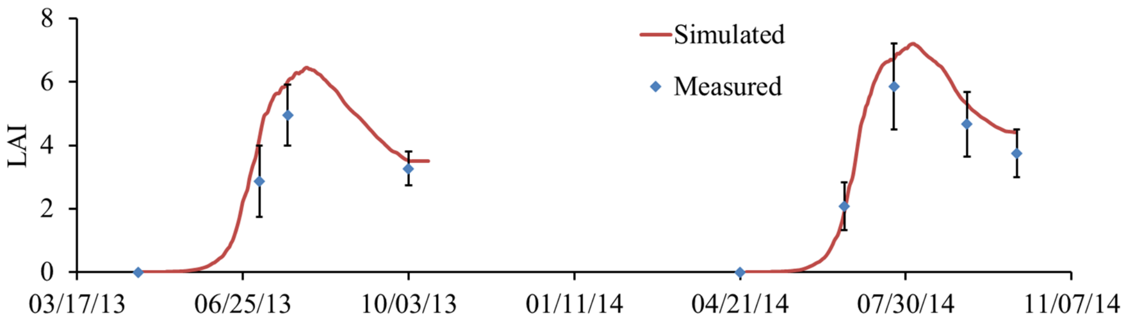

Figure S15. Comparison of measured and simulated leaf area index (LAI) under N168 treatment in loamy clay soil.

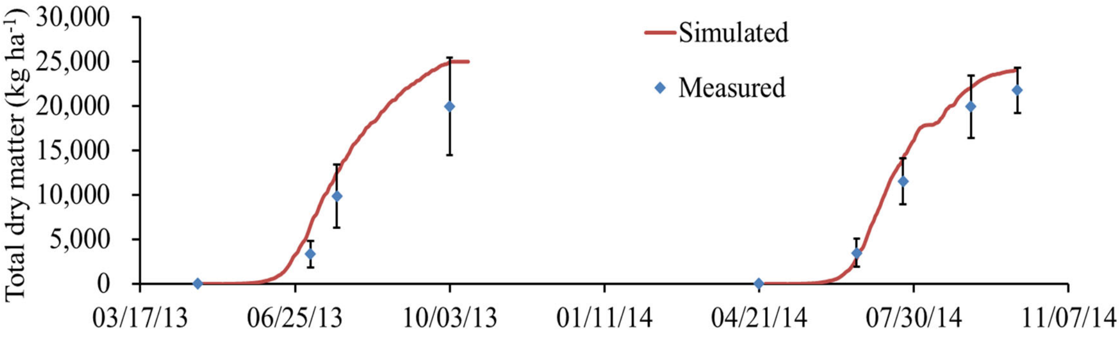

Figure S16. Comparison of measured and simulated total dry matter (TDM) under N168 treatment in loamy clay soil.

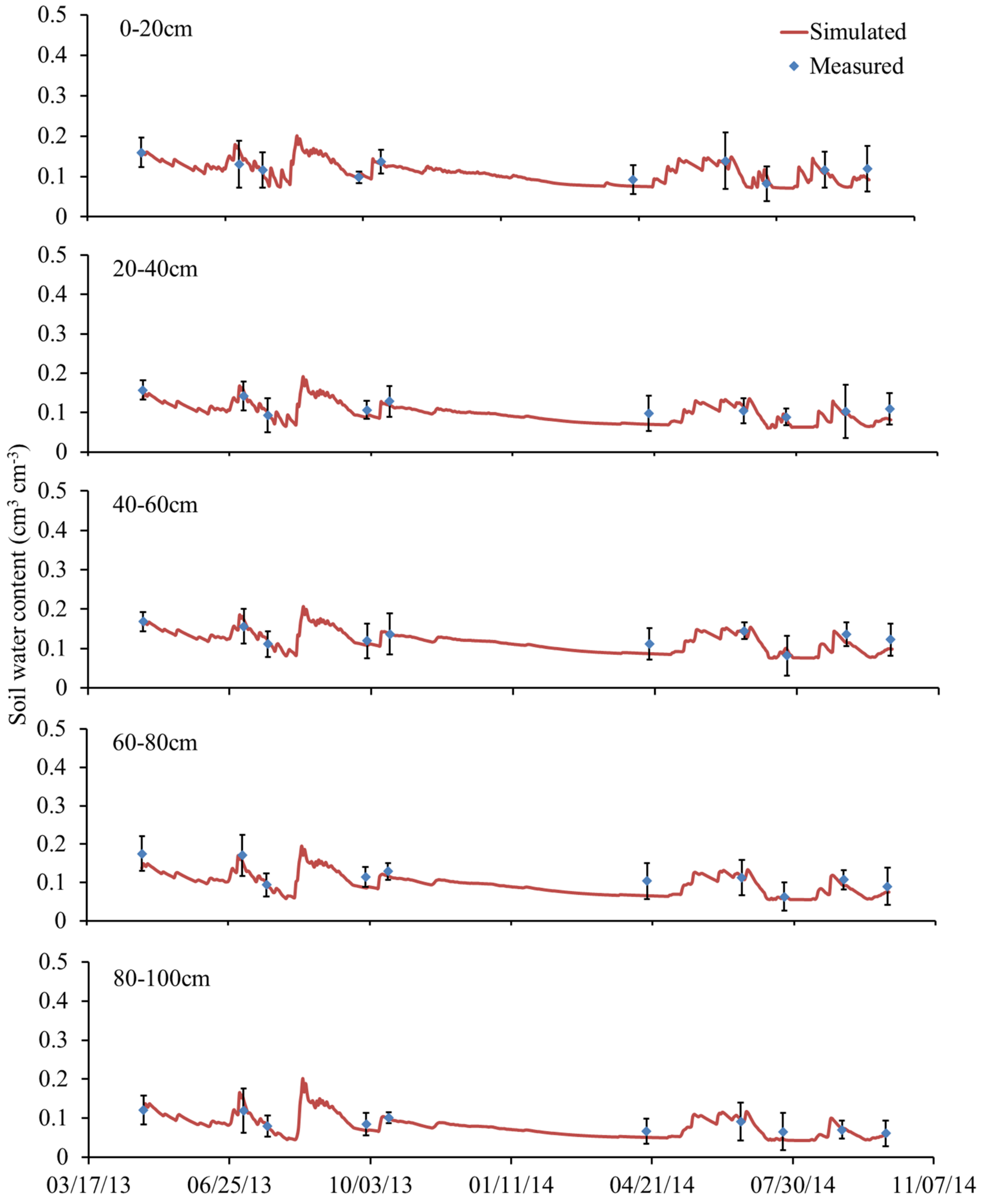

Figure S17. Comparison of simulated and measured soil water content at different depths under N168 treatment in Sandy loam soil.

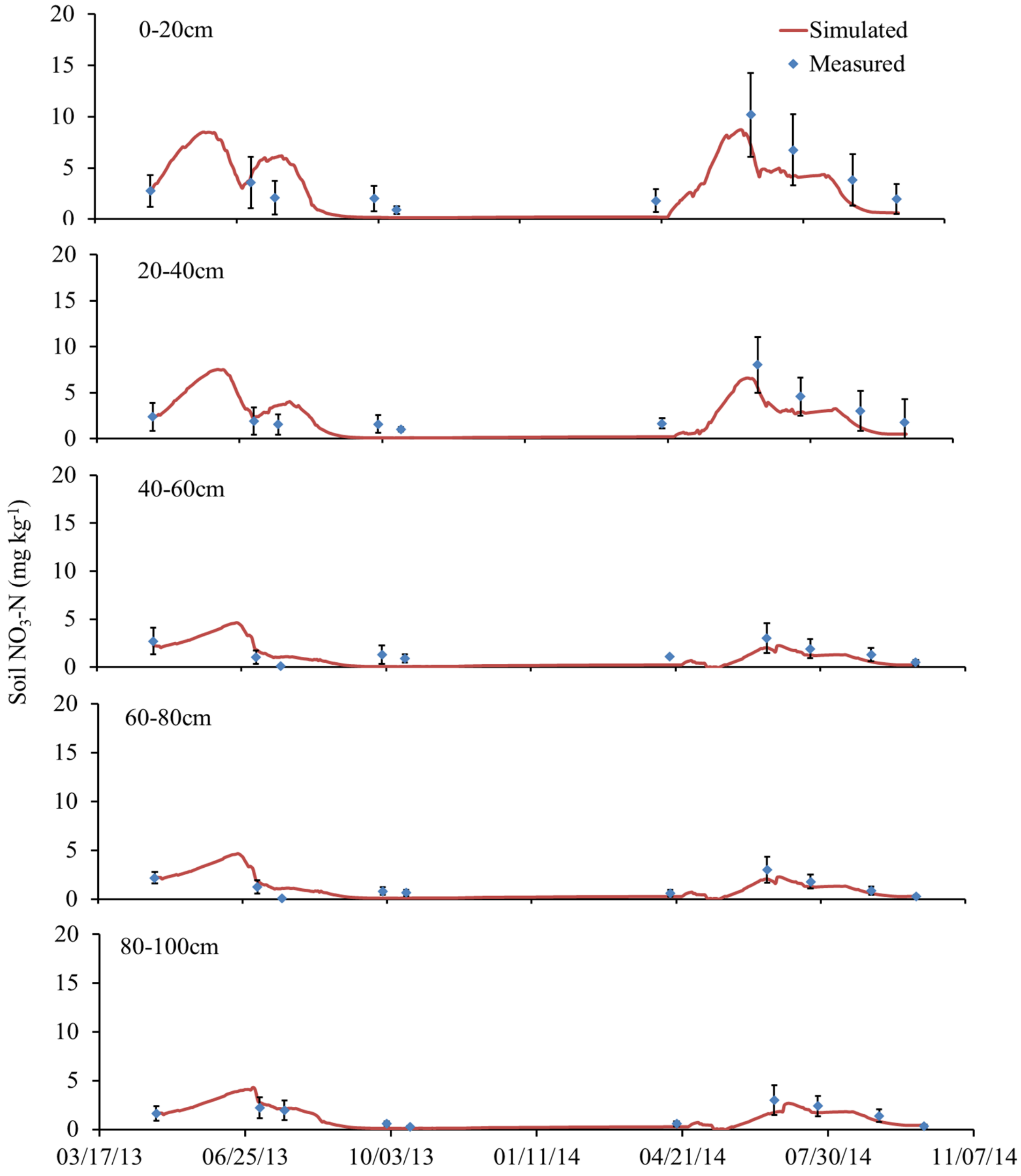

Figure S18. Comparison of simulated and measured soil nitrate concentration at different depths under N168 treatment in Sandy loam soil.

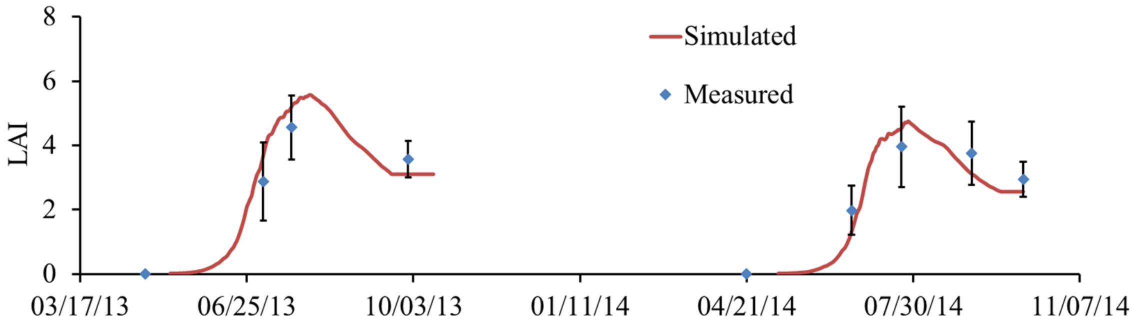

Figure S19. Comparison of measured and simulated leaf area index (LAI) under N168 treatment in Sandy loam soil

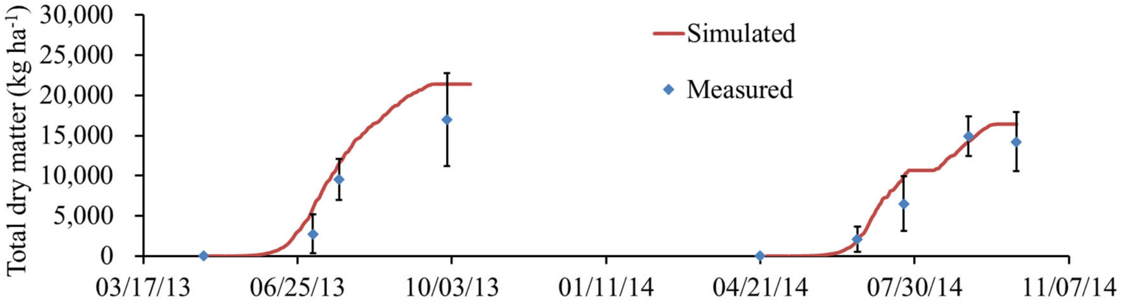

Figure S20. Comparison of measured and simulated total dry matter (TDM) under N168 treatment in Sandy loam soil.

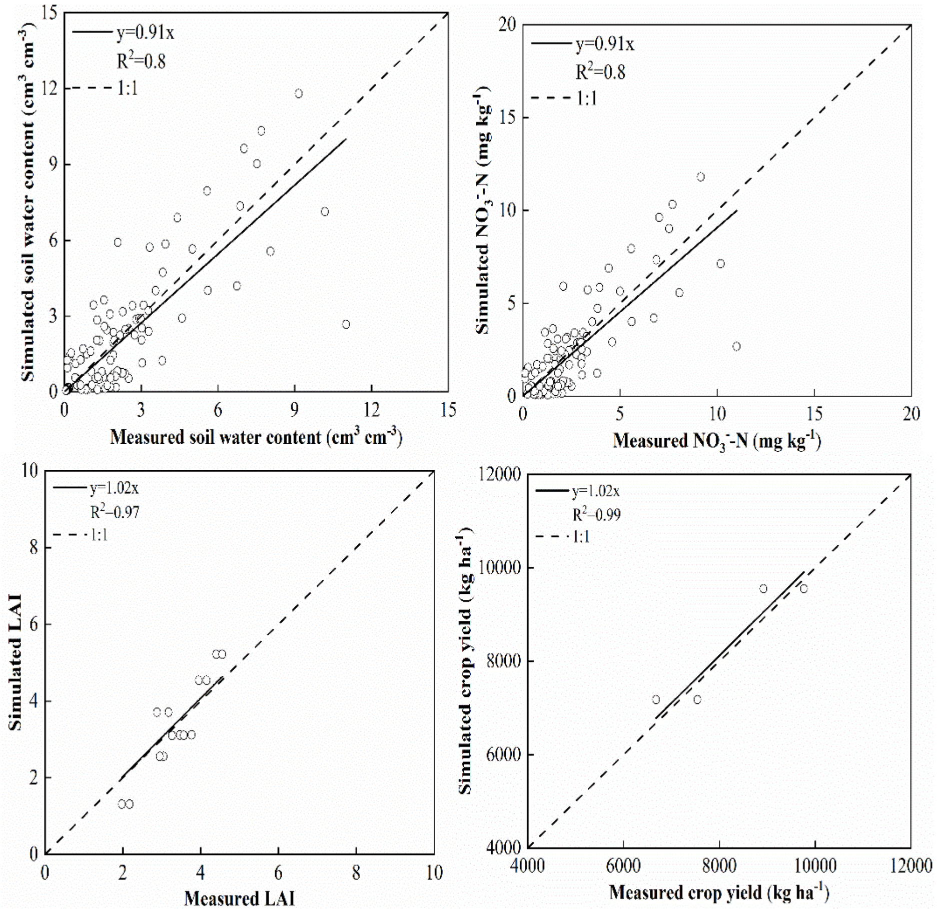

Figure S21. The relationship between measured and simulated soil water contents, nitrate concentrations, crop yields, and LAI under the validation treatments in Sandy loam soil.

Table S1. Soil physical-chemical properties for soil profile in two experimental sites.

Site	Soil organic matter g kg^{-1}	total N g kg^{-1}	pH	Alkaline-N mg kg^{-1}	Olsen-P mg kg^{-1}	Available-K mg kg^{-1}
Fujiajie	13.2	1.01	6.71	58.8	13.6	43
Sankeshu	25.3	1.69	6.16	128.2	43.9	122

Table S2. Analysis of significant different for yield and nitrogen uptake under different N rates for two textural soils in 2013 and 2014.

Soil	Pattern	Yield (kg ha^{-1})		N uptake (kg ha^{-1})	
		2013	2014	2013	2014
Sandy loam	N168	8922±11b	6677±299a	136±2b	120±2a
	N240	9767±172a	7541±368a	178±7a	150±26a
	N312	9826±249a	6808±421a	172±2a	167±24a
	average	9505±170b	7008±205b	162±6b	146±13b
Loamy clay	N168	9947±343a	11923±725a	197±7a	186±10a
	N240	11478±58a	12576±228a	195±4a	203±12a
	N312	10895±421a	11822±639a	201±10a	211±6a
	average	10439±223a	12107±349a	197±4a	200±6b

Note: means between treatments followed by different letters indicated a significant difference at $P<0.05$
